# Public Opinions on Strategies for Managing Stray Cats and Predictors of Opposition to Trap-Neuter and Return in Brisbane, Australia

**DOI:** 10.3389/fvets.2018.00290

**Published:** 2019-02-18

**Authors:** Jacquie Rand, Gina Fisher, Kate Lamb, Andrea Hayward

**Affiliations:** ^1^School of Veterinary Science, University of Queensland, Brisbane, QLD, Australia; ^2^Australian Pet Welfare Foundation, Brisbane, QLD, Australia

**Keywords:** trap neuter return, urban stray, cats, sterilize, euthanasia

## Abstract

A survey of Brisbane residents was undertaken to investigate community attitudes toward urban stray cats and their management. Surveys were distributed to 84 medical and dental practices across Brisbane City, and were completed by 305 patients and staff. Practices were targeted to achieve a sample of respondents from a representative distribution of socioeconomic backgrounds. After being informed about trap, neuter, and return (TNR) programs for management of urban stray cats, most respondents (79%), chose TNR as their preferred management strategy, while a lesser proportion (18%) expressed a preference to continue the current Brisbane City Council lethal control program (catching and culling ~1,000 cats annually), and 3.4% selected to leave the cats alone. Differences in beliefs and attitudes toward urban stray cats as a function of demographic variables were investigated. Statistical analyses indicated that respondents who were male, older, non-cat owners, those who believed euthanasia of stray cats was humane, and that urban stray cats spread disease to humans were significantly more likely to express a preference for lethal control, as opposed to non-lethal population management. Based on these findings, we recommend that information is disseminated to mitigate these concerns or negative beliefs, where warranted. Ultimately, findings from this study demonstrate that current Queensland legislation does not reflect public views and opinions on stray cat management and should be reviewed. Formal research evaluating the efficacy of TNR programs for urban stray cats in Australia would be in the public interest.

## Introduction

Like many countries, in urban areas of Australia, unowned cats result in complaints to local government bodies responsible for animal management, and result in costs associated with mitigating these complaints. Complaints relating to free-living cats stem from nuisances caused by fighting, soiling property, and the perception of threats to human and pet health (1, 2). In addition, there are concerns about the welfare of urban stray cats themselves (3–6), but there are also concerns about the ecological impact of stray cats killing birds, small mammals, and other suburban native wildlife (7–9). As such, effective interventions are needed to manage the stray cat population, which in turn will reduce costs associated with mitigating such complaints. Australia's urban stray cat population is estimated at ~1.2–2 million (10). In cities, the number of stray cats is estimated to be 60–100 per 1,000 human residents (11–13), but may be higher or lower depending on the location (14). Approximately 85% of cats admitted to Australian municipal animal facilities and 50–70% admitted to animal welfare shelters are urban strays, and on average 48–56% of all impounded cats across Australia are euthanized (12, 15–17). The resulting large number of euthanized kittens and cats, mostly young and healthy, produce perpetration-induced posttraumatic stress in many workers directly involved with their euthanasia (18). Workers also experience other mental and physical health issues such as depression, substance abuse, high blood pressure, sleeplessness, and increased risk of suicide (18–20).

Over time cat numbers can be reduced by culling, or, by preventing reproduction. In open populations, culling at a rate able to achieve population control requires removing 30–50% of the cat population every 6 months for ~10 years (21), which is beyond most local government budgets, unlikely to be acceptable in the community, and would certainly lead to an increase in the mental health issues already prevalent in those tasked with euthanizing the cats (19). In a city with a population of 1 million (approximate size of Brisbane), using modeling that specifies a culling rate of 40% for a population of 60,000 stray cats, it is estimated that 40,000 cats would need to be killed in the first year alone to effectively reduce the stray cat population (21). In contrast, the low-level culling of stray cats (2–5% annually) that is typically used by municipalities (10, 17) is ineffective at decreasing the urban stray cat population, and can paradoxically encourage population rebound, or even growth, due to an influx of new stray cats to the area, and increased juvenile survival due to less competition for resources (22–24).

Culling programs may also be highly unfavorable with members of the public. Overseas, it has been found that lethal control methods are strongly opposed, especially by cat owners (25–29). Likewise, it has been strongly opposed by those who have formed an attachment with strays in their neighborhood or who exhibit “semi-ownership” bonds with these animals (30). Lethal control methods without community support have even resulted in sabotage of the program (31). Performing the level of culling required to render lethal programs effective could significantly worsen mental health issues already prevalent in animal management employees (18), and may likely be met with significant community backlash.

An alternative to culling is to trap, neuter, and return (TNR) stray cats to the location in which they were originally found. This method has been shown in both the USA and Australia to effectively reduce cat numbers in targeted urban and periurban areas (10, 14, 17, 31–35), reduce the intake and subsequent euthanasia in shelters, and reduce cat-related complaints (31, 32, 36–39). Thus, it may be a more effective alternative to the current low level culling of urban stray cats, more humane to the animals, and relieve strain and burden from shelter facilities and their workers. Although some earlier studies reported that cat numbers did not decrease with TNR, typically this was because adequate sterilization rates were not achieved and/or immigrant and dumped cats were not quickly managed by sterilization and the adoption of socialized cats (40–42). For either trap and kill or TNR to result in a reduction in cat numbers over time, more than 50% of the population must be culled or sterilized annually (21, 43–45). Although some modeling studies suggest that trap and kill reduces cat numbers faster than TNR (21, 43), the magnitude of the culling is beyond the budgets and tolerance of most communities. Of note, there are no published studies from Western countries reporting successful trap and kill programs for cats in either a zipcode or city, in contrast to a number of effective large-scale TNR programs reported in the literature (14, 38). Based on current international literature, when conducted using best practice, TNR is an efficacious and viable method in which to manage stray cats in urban communities. It reduces strain on shelters by reducing cat intake, and support from the community typically helps to defray government costs. TNR programs that actively place the more sociable stray cats and kittens up for adoption achieve a quicker initial reduction in cat numbers (10). Potential for disease transmission to humans, pets and wildlife is also likely reduced because fighting and roaming behaviors in sterilized cats are less frequent than in entire (i.e., non-sterilized) cats, and there are fewer kittens to shed parasite eggs or oocysts (toxoplasma) compared to trap and kill programs (46–48).

Management of urban stray cats is an emotive issue because of the wide diversity of public perceptions about stray cats and differences in the way people interact with these animals. To date, the majority of TNR research has been conducted internationally, and data are lacking in Australia with regards to how the general public prefers unowned urban cats to be managed. Brisbane is the capital city for the state of Queensland in Australia, and the Brisbane City Council's (BCC)^1^ local government area has a population of ~1.2 million—it is roughly equivalent to the population of Tasmania, ACT, and the Northern Territory combined. As well as this, there is a high diversity of demographic and socioeconomic characteristics (49). This size makes it an ideal Australian city to study a variety of opinions on stray cats.

The BCC has an active cat trapping program targeted to locations of community complaints and stray cat sightings, and the current program has a target of 1,000 cats per year, most of which are killed (50). An additional ~700 are euthanized annually in the municipal pound and local welfare agency shelter, representing a total cull rate of ~2.5% of the estimated free-living cat population (unpublished data, author JR). Cat legislation in Queensland is very restrictive and disallows the possibility of using TNR. Under the *Biosecurity Act of 2014*^2^, and *Land Protection (Pest and Stock Route Management) Act 2002*,^3^ no distinction is made between urban strays and truly feral cats in remote areas which get no support from humans for food or shelter, despite fundamental differences between these groups of cats. Under Queensland legislation, both are classed as non-domestic cats, with only owned cats classed as domestic. The acts stipulate that non-domestic cats “must not be moved, fed, given away, sold, or released into the environment without a permit”. Due to this legislation, many TNR activities in Queensland and other Australian states are conducted unofficially by rescue organizations and volunteers (10).

Assessing the level of public support for TNR is vital to obtaining supporting evidence for governments interested in making legislative changes. Furthermore, knowledge of public support for non-lethal control methods of urban stray cats would facilitate more formal research into the efficacy of TNR in an Australian context. Overseas studies show that the majority of people surveyed prefer non-lethal cat management programs in comparison to culling (2, 28, 51, 52). However, Brisbane is one of the most biodiverse capital cities in Australia^1^, and substantial media has focused on the negative impact of cats on native wildlife (53). Therefore, it is unknown if residents of Brisbane largely support current lethal methods of cat control in the city, or have similar attitudes to residents overseas who prefer non-lethal control.

The aims of this study were to determine the attitudes of Brisbane city residents toward urban stray cats and factors which affect respondents' preferences for stray cat management methods. In doing so, we aim to identify the most salient concerns about urban stray cats held by those in opposition to TNR, and identify the most effective method to mitigate such concerns where warranted. Finally, we aim to provide evidence of the need to facilitate formal research into the efficacy of TNR as an alternative to current stray cat management methods in Australia.

## Materials and Methods

### Study Design Overview

A cross-sectional study was conducted with adult residents of the BCC area recruited from those attending selected medical and dental practices, and participants of a community group between 17th August 2017 and January 30th 2018. The Australian Bureau of Statistics (ABS) index of relative socioeconomic advantage and disadvantage (SEIFA score) values (54) as at 2011 for each of the 71 postcode areas in the BCC area were identified. One quarter of the postcodes were placed in each of four strata based on their SEIFA score. We then randomly selected 5 postcodes from each of the four socioeconomic quartiles with replacement (i.e., the same postcode could be selected more than once) using probability in proportion to size sampling (PPS), where postcodes having higher populations were proportionally weighted to have a higher chance of being selected. Resident populations as at 2011 were used.

We then identified all medical practices within each of the selected postcodes, allocated these with a number and used a random number generator to select one practice from each postcode (except for two postcodes selected twice in which case two practices were selected). Practice managers from each clinic were called to gain permission to leave the survey forms within their clinic's waiting room, and were asked if reception staff could inform patients of the survey's existence, which could be completed while waiting for their appointment. Reception staff were asked if they could encourage a 50/50 male: female ratio of respondents. Practices that declined to be involved were removed from the list and the random number generator was used to select another practice from that postcode. Where all medical practices in selected postcode areas declined to participate, replacement postcodes were randomly selected from the same socioeconomic quartile as described above. All random selections were made using Microsoft Excel's RANDBETWEEN function. Practices that granted permission to conduct the survey were delivered blank copies of the survey. Completed surveys were collected from the practice 2–4 weeks later. To increase the number of completed surveys, dental practices closest to the medical practices were then later included, as was a community group involved with restoration of a waterway (catchment group). Surveys were completed from 30 medical practices, 54 dental practices and the catchment group (15 surveys only).

The survey contained four groups of questions concerning general information on respondents and their pet ownership history, and residents' attitudes and interactions with urban stray cats (assessed by responding to statements with a five-point Likert scale, with 1 denoting *strongly disagree* and 5 denoting *strongly agree*). Preferences for the management of strays before and after being provided with information about TNR were assessed via the selection of one of three discrete options. Attitudes toward a trial of TNR in their community was assessed via responses to a statement using the same Likert scale described, and the selection of discrete answers provided in response to the question. The full survey is available in the Appendix (Table A1 in Supplementary Material). For demographic questions, age groupings were based on ABS groupings to allow comparison with the Australian population. Education level was classed on a scale between 1 and 4 based on respondents' answers to “what is your highest level of education?” in line with the Australian Qualifications Framework (55).

Questions pertaining to attitudes about urban stray cats were formulated in response to commonly reported complaints and concerns in communities cited in prior literature (1–3, 5, 6, 8). A portion of the questions were adapted from a prior survey [items 4 and 5; (3)]. A small pilot study of 17 participants was performed to gain feedback on the clarity of questions, and those deemed unclear were subsequently reworded and tested again. This was performed prior to printing and distributing the surveys for the main study. Data from the pilot study were not included in the study results.

### Statistical Analyses

Statistical analyses of the 305 questionnaires aimed to determine what factors may be associated with negative attitudes toward urban strays, and factors associated with the preference for lethal as opposed to non-lethal urban stray population management. Firstly, a series of chi-square tests were conducted to examine whether there were differences in the pattern of responses for key questionnaire items based on demographic variables. Independent variables were categorical, and included age (above vs. below the modal age), gender (male vs. female), pet ownership status (owner vs. non-owner), cat ownership status (owner vs. non-owner), and respondents' awareness of strays (i.e., aware vs. unaware of strays). The dependent variable in each test was ordinal in nature and consisted of the level of agreement with the given questionnaire item from 1 (strongly disagree) to 5 (strongly agree). Cross tabulations between demographic variables and agreement level were analyzed (all tables are available in the Appendix in Supplementary Material). As per the requirements of a chi-square analysis of association, no table cells had <1 observation, and at least 80% of all cells had more than 5 observations (56).

Secondly, a logistic regression was performed to determine whether certain demographic variables (education level, gender, cat ownership status, age, and SEIFA score) were predictive of respondents' preferences for managing stray cat populations (lethal vs. non-lethal). A separate logistic regression was performed to determine whether responses to certain attitudinal measures (belief that cats spread diseases to humans or pets, the belief that urban strays reduce native birds or small animals, and the belief that euthanasia would be more humane than leaving an urban stray cat in their environment) were predictive of lethal vs. non-lethal preferences for stray cat management strategies. For each statistical test, only respondents who had provided a valid response to all items in the model were included. The sample size of both logistic regressions adhered to the established rule of thumb that regression or cox analyses require a minimum of 10 observations per predictor (57), or in the case of a binary logistic regression, a minimum of 5–9 observations per predictor (58).

## Results

### Respondent Demographics

Completed surveys were obtained from 305 respondents. On average, only 1.6% of survey items were left unanswered by respondents (range = 0–5.3%, *SD* = 1.3%), demonstrating a good level of engagement with surveys. Seventy-percent of participants were female, 27% male, and 1% identified as “other” (2% of respondents did not provide a response). Respondents specified which ABS age bracket they belonged to. The median age bracket was 35 to 39 years of age, and the modal age bracket was 18–24 years of age, with 22.2% of respondents coming from this bracket. Most respondents reported being born in Australia (73%), however, 20 other countries of birth were represented. The next most commonly reported places of birth were New Zealand (5%) and the United Kingdom (5%). The majority of respondents held a university degree or graduate diploma (47%), and a large proportion posessed a vocational certificate or secondary school certificate (28%). The majority of respondents owned a pet (76%), with cats being the most common (56%), followed by dogs (52%), birds (10%), reptiles (1%), and fish (1%). Of cat owners, most had a single cat, but 45% had two or more. The majority of cat-owners reported that all cats owned were microchipped (89%) and sterilized (93%). Respondents came from 34 of the 71 postcode areas within the Brisbane metropolitan area, thereby representing 48% of the total postcodes. The average SEIFA score of socioeconomic advantage and disadvantage for respondents was 1,054 (*SD* = 83), which was close to the average for the BCC local government area of 1,052.

### Respondents' Awareness of Strays and Feeding Behavior

Less than half (43%) the respondents reported that they were aware of stray cats in their area, while 57% were unaware. Stray cats were observed in a wide variety of locations, with the most common being private residences, alleyways, and commercial businesses (i.e., eateries and shops; Table 1).

**Table 1:** Locations of reported stray cat sightings and associated proportion of total sightings.

**Sighting location**	**Frequency**	**Proportion of total sightings (%)**
Private residences	44	20.5
Commercial businesses	33	15.3
Alleyways	33	15.3
Suburban parks	27	12.6
Industrial areas	22	10.2
Vacant blocks	19	8.8
Schools	18	8.4
Train stations	12	5.6
Government housing	7	3.3

Fifteen percent of respondents reported feeding urban stray cats. Of these respondents, 18% fed strays on a daily basis (3% of all respondents), 11% on a weekly basis, 28% on a monthly basis, and 43% on a yearly basis. Cat feeders were represented in every age bracket, with the median being the 30 to 34 years of age, and the mode being the 18–24 years of age bracket (representing 29.5% of cat feeders). Similar proportions of females (14.4%) and males (13.4%) reported feeding urban stray cats. Many cat feeders did not own a cat (38.6%), but most were cat owners (61.4%); cat feeders accounted for 9.7% of all non-cat owners, and 20.8% of all cat-owners.

### Perceptions Regarding Nuisance Behaviors of Urban Stray Cats

More participants agreed (i.e., either selected agree or strongly agree) than disagreed (i.e., selected disagree or strongly disagree) that stray cats caused a nuisance by urinating and defecating in people's gardens (45.3 vs. 28.1%), and are annoying because they fight and make loud noises (46.2 vs. 25.8%; Table 2). However, many respondents did not hold an opinion and expressed a neutral attitude toward items (27–44%). Older respondents and those who reported being aware of strays were more inclined to agree with the nuisance behavior items than younger respondents and those that were not aware of strays (Table 2 and Table A2 in Supplementary Material). Additionally, cat-owners demonstrated less agreement toward both nuisance behavior items than non-cat owners, and pet-owners demonstrated less agreement with the statement that cats caused a nuisance by defecating and urinating than non-pet owners.

**Table 2:** Response distributions for survey items pertaining to nuisance behaviors of stray cats, and chi-square tests for differences in response distributions as a function of demographic variables.

**Survey item (number of valid responses for item)**	**Response proportions as % and (frequencies)**	**Demographic variables tested (number of respondents in model)**	**Pearson's Chi-Square statistic and degrees of freedom**	***p*-values**
Cause a nuisance by urinating and defecating in people's gardens (302)	SD = 11.9 (36)	Gender (294)	$\chi_{\left(4\right)}^{2}$ = 3.57	*p* = 0.468
	D = 16.2 (49)	**Age (297)**	$\chi_{\left(4\right)}^{2}$ **= 19.87**	***p*** = 0.001^***^
	N = 26.5 (80)	**Own-pet (298)**	$\chi_{\left(4\right)}^{2}$ **= 10.79**	***p*** = 0.029^*^
	A = 24.8 (75)	**Own-cat (302)**	$\chi_{\left(4\right)}^{2}$ **= 41.81**	***p*** < 0.001^***^
	SA = 20.5 (62)	**Aware of Strays (298)**	$\chi_{\left(4\right)}^{2}$ **= 34.18**	***p*** < 0.001^***^
Are annoying because they fight and make loud noises (303)	SD = 10.9 (33)	Gender (295)	$\chi_{\left(4\right)}^{2}$ = 7.09	*p* = 0.131
	D = 14.9 (45)	**Age (298)**	$\chi_{\left(4\right)}^{2}$ **= 24.21**	***p*** < 0.001^***^
	N = 28.1 (85)	Own-pet (299)	$\chi_{\left(4\right)}^{2}$ = 6.33	*p* = 0.176
	A = 25.7 (78)	**Own-cat (303)**	$\chi_{\left(4\right)}^{2}$ **= 34.01**	***p*** < 0.001^***^
	SA = 20.5 (62)	**Aware of Strays (299)**	$\chi_{\left(4\right)}^{2}$ **= 14.10**	***p*** = 0.007^**^
Spread diseases to humans (301)	SD = 16.3 (49)	Gender (293)	$\chi_{\left(4\right)}^{2}$ = 7.65	*p* = 0.105
	D = 22.3 (67)	Age (296)	$\chi_{\left(4\right)}^{2}$ = 4.01	*p* = 0.405
	N = 43.5 (131)	**Own-pet (297)**	$\chi_{\left(4\right)}^{2}$ **= 16.81**	***p*** = 0.002^**^
	A = 8.6 (26)	**Own-cat (301)**	$\chi_{\left(4\right)}^{2}$ **= 56.66**	***p*** < 0.001^***^
	SA = 9.3 (28)	**Aware of Strays (297)**	$\chi_{\left(4\right)}^{2}$ **= 15.69**	***p*** = 0.003^**^
Spread diseases to owned pets (299)	SD = 8.7 (26)	Gender (291)	$\chi_{\left(4\right)}^{2}$ = 5.12	*p* = 0.275
	D = 8.7(26)	Age (294)	$\chi_{\left(4\right)}^{2}$ = 3.85	*p* = 0.426
	N = 34.4 (103)	Own-pet (295)	$\chi_{\left(4\right)}^{2}$ = 6.80	*p* = 0.147
	A = 31.8 (95)	**Own-cat (299)**	$\chi_{\left(4\right)}^{2}$ **= 18.61**	***p*** = 0.001^***^
	SA = 16.4 (49)	**Aware of Strays (295)**	$\chi_{\left(4\right)}^{2}$ **= 15.37**	***p*** = 0.004^**^

*SD, strongly disagree; D, disagree; N, neutral; A, agree; SA, strongly agree*.

* Significant at the < 0.05 level;

** Significant at the ≤ 0.01 level;

*** *Significant at the ≤ 0.001 level. Response distributions associated with significant chi-square results are displayed in plots under the explanation of findings for the given items. For simplicity, descriptive statistics for non-significant results are not reported. Bold indicates variables with significantly different response distributions at P < 0.05*.

### Perceptions Regarding Spread of Disease

**Figure 1:**
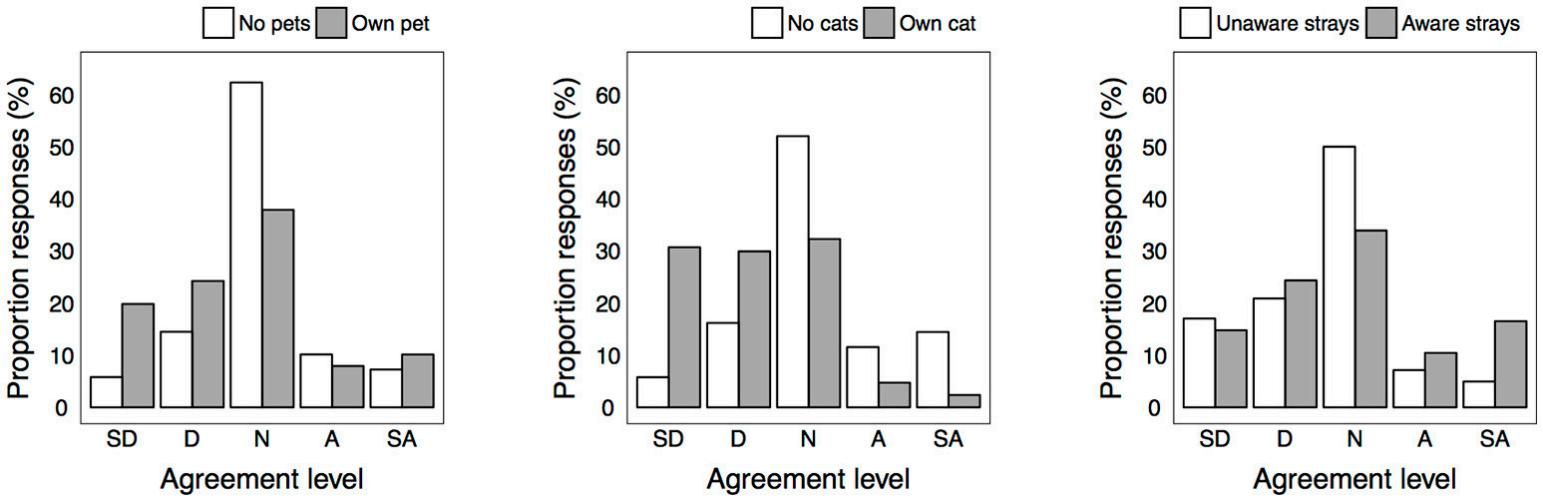
Level of agreement for statement “urban stray cats spread diseases to humans” between significantly different groups. SD, strongly disagree; D, disagree; N, neutral; A, agree; SA, strongly agree.

More respondents disagreed (38.6%) than agreed (17.9%) that stray cats spread diseases to humans. Cat and pet-owners were more inclined to disagree or have a neutral opinion than respondents that owned no pets (Figure 1, Table 2, and Table A1 in Supplementary Material). Contrastingly, those who were aware of strays were more inclined to agree that cats spread diseases to humans than those unaware of strays. More respondents agreed (48.2%) than disagreed (17.4%) that stray cats spread diseases to owned pets. Again, cat-owners appeared to express more disagreement than non-cat owners, and those who were aware of strays expressed more agreement than respondents unaware of strays.

### Perceptions Concerning Effects on Wildlife

**Figure 2:**
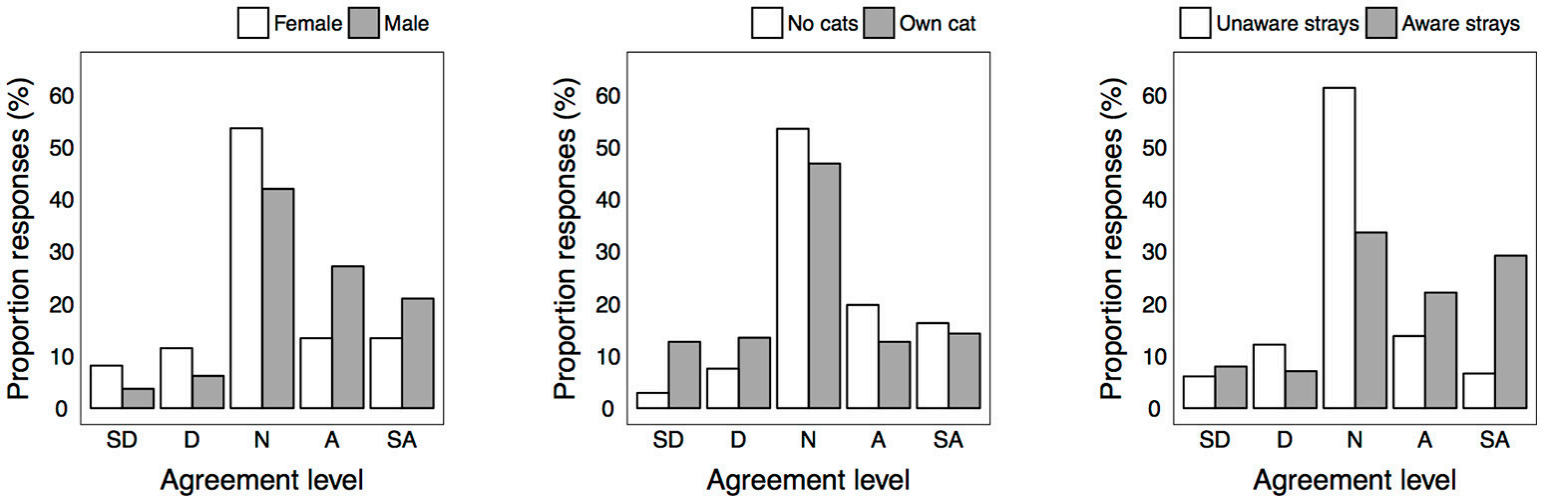
Level of agreement for statement “urban stray cats decrease the number of native birds in my suburb” for each significantly different group.

Respondents' views on the impact of urban stray cats on wildlife were varied, but more respondents agreed that urban stray cats decreased the number of native birds in their suburb compared to those that disagreed (31.8 vs. 18.3%; Table 3). In addition, more respondents agreed that urban stray cats decreased the numbers of small native animals compared to those who disagreed (32.9 vs. 19.0%). Females and cat owners expressed less agreement with the ecological impact items than males or non-cat owners (Figure 2 and Table A2 in Supplementary Material). Those that were aware of strays expressed more agreement with ecological impact items than those who were not aware.

**Table 3:** Response distributions for survey items pertaining to stray cats' ecological impact and chi-square tests for differences in response distributions as a function of demographic variables.

**Survey Item (number of valid responses for item)**	**Response proportions as % and (frequencies)**	**Demographic variables tested (number of respondents in model)**	**Pearson's Chi-Square statistic and degrees of freedom**	***p*-values**
Urban stray cats have decreased the number of native birds in my suburb (302)	SD = 7.0 (21)	**Gender (294)**	$\chi_{\left(4\right)}^{2}$ **= 14.59**	***p*** = 0.006^**^
	D = 11.3 (34)	Age (297)	$\chi_{\left(4\right)}^{2}$ = 4.81	*p* = 0.308
	N = 50.0 (151)	Own-pet (298)	$\chi_{\left(4\right)}^{2}$ = 7.22	*p* = 0.125
	A = 16.6 (50)	**Own-cat (302)**	$\chi_{\left(4\right)}^{2}$ **= 16.63**	***p*** = 0.002^**^
	SA = 15.2 (46)	**Aware of Strays (298)**	$\chi_{\left(4\right)}^{2}$ **= 37.99**	***p*** < 0.001^***^
Urban stray cats have decreased the number of small native animals in my suburb (301)	SD = 6.0 (18)	**Gender (293)**	$\chi_{\left(4\right)}^{2}$ **= 15.86**	***p*** = 0.003^**^
	D = 13.0 (39)	Age (296)	$\chi_{\left(4\right)}^{2}$ = 4.39	*p* = 0.356
	N = 48.2 (145)	Own-pet (297)	$\chi_{\left(4\right)}^{2}$ = 4.82	*p* = 0.306
	A = 17.3 (52)	**Own-cat (301)**	$\chi_{\left(4\right)}^{2}$ **= 19.44**	***p*** = 0.001^***^
	SA = 15.6 (47)	**Aware of Strays (297)**	$\chi_{\left(4\right)}^{2}$ **= 38.11**	***p*** < 0.001^***^

** Significant at the ≤ 0.01 level;

*** *Significant at the ≤ 0.001 level. Response distributions associated with significant chi-square results are displayed in plots under the explanation of findings for the given items. Bold indicates variables with significantly different response distributions at P < 0.05*.

### Caring and Humane Attitudes to Urban Stray Cats

**Figure 3:**
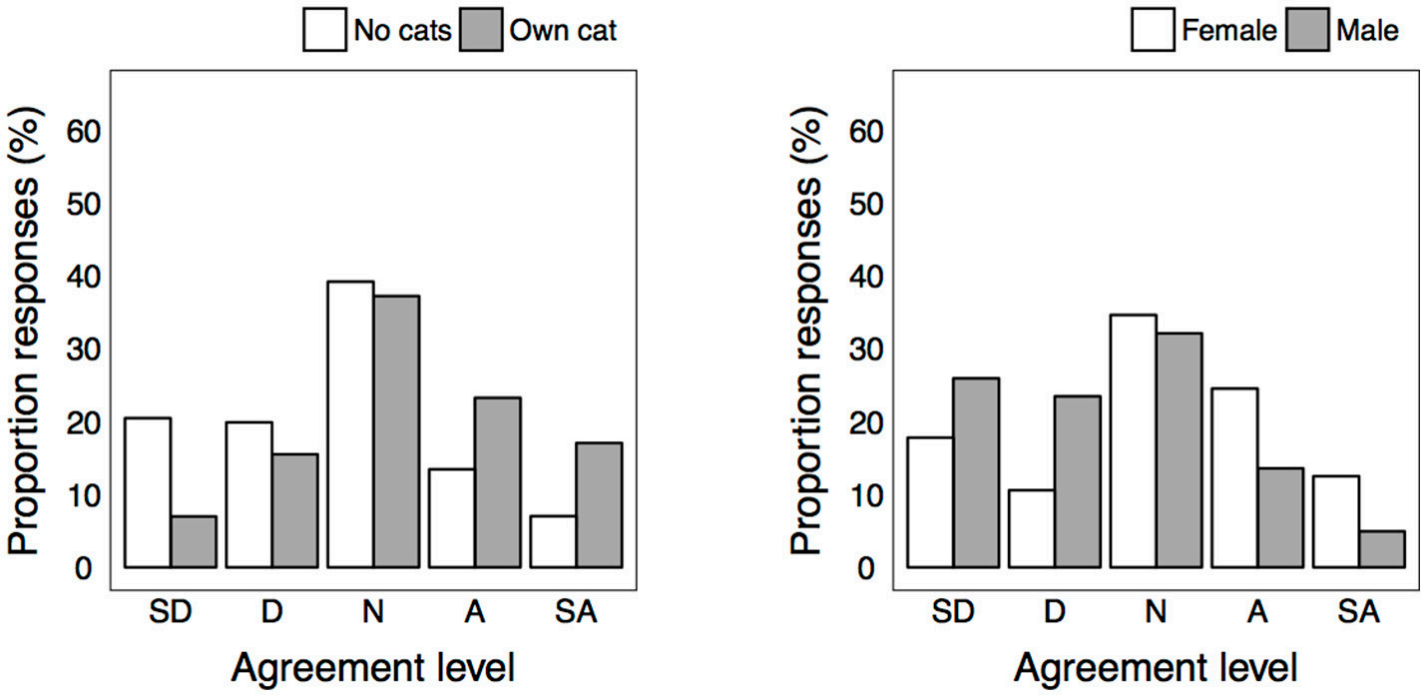
Level of agreement for statement “seeing a healthy stray cat would make me feel good” for each significantly different group.

**Figure 4:**
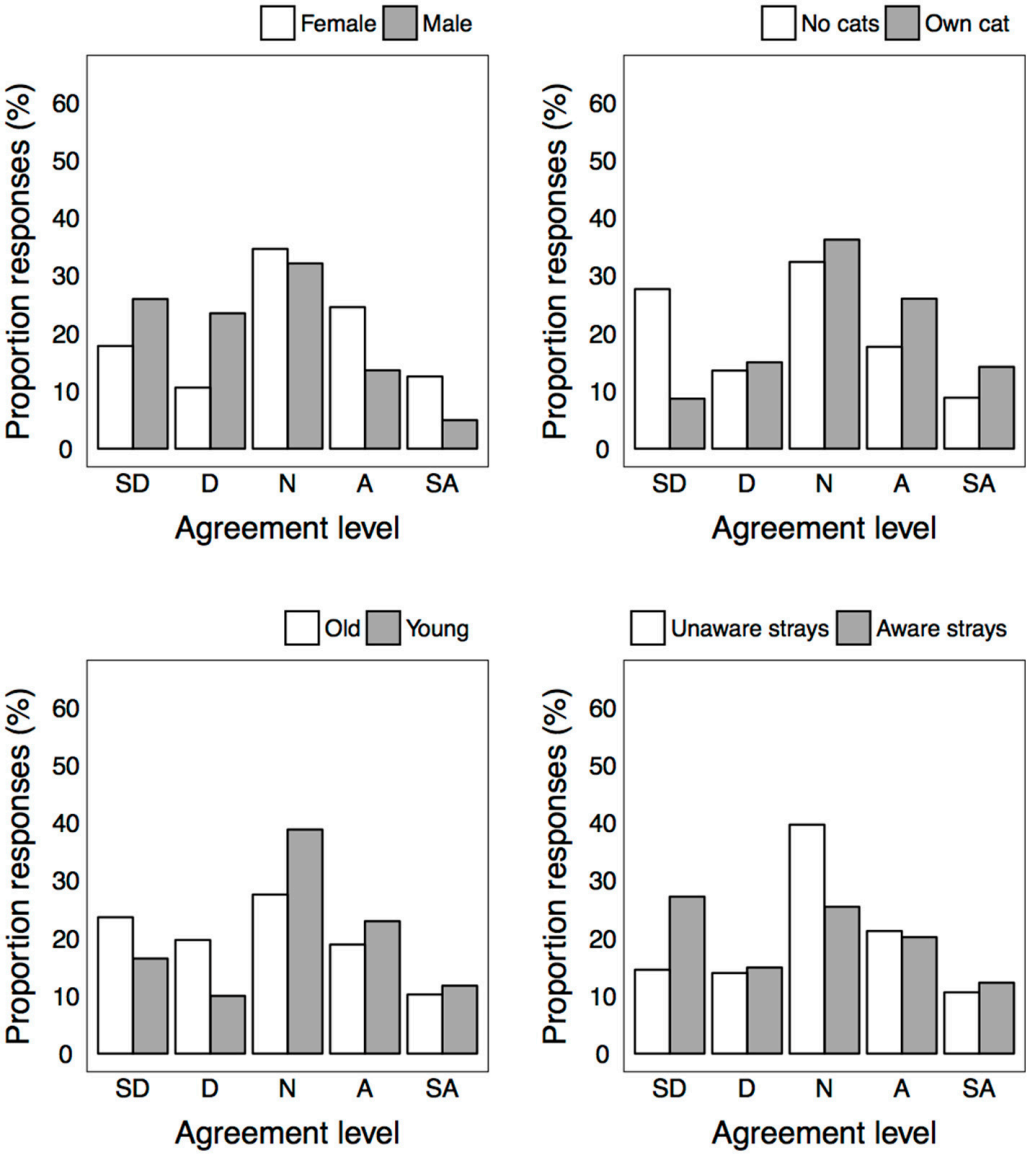
Level of agreement for statement “feeding a healthy stray cat would make me feel good” for each significantly different group.

Very few respondents were of the view that urban stray cats had a good life (5.4%), with just over half disagreeing (51.5%), and a large proportion neither agreeing nor disagreeing (43.1%). Responses did not differ based on any demographic factors (Table 4). Respondents' agreement as to whether seeing a healthy stray cat, or feeding a stray cat would make them feel good varied substantially, and many respondents neither agreed nor disagreed (34.1 and 38.3%) (Figures 3, 4). Cat-owners expressed more agreement with both items, while respondents who reported being aware of strays expressed more disagreement with both items (Figure 3). Interestingly, the proportion of responses for the statement “feeding a stray cat would make me feel good” also differed depending on gender and age. Males and older participants appeared to express more disagreement with the item than did females or younger respondents.

**Table 4:** Response distributions for survey items pertaining to welfare of stray cats and chi-square tests for differences in response distributions as a function of demographic variables.

**Survey Item (number of valid responses for item)**	**Response proportions as % and (frequencies)**	**Demographic variables tested (number of respondents in model)**	**Pearson's Chi-Square statistic and degrees of freedom**	***p*-values**
Urban stray cats have a good life (297)	SD = 21.2 (63)	Gender (289)	$\chi_{\left(4\right)}^{2}$ = 3.13	*p* = 0.537
	D = 30.3 (90)	Age (292)	$\chi_{\left(4\right)}^{2}$ = 1.32	*p* = 0.858
	N = 43.1 (128)	Own-pet (293)	$\chi_{\left(4\right)}^{2}$ = 2.40	*p* = 0.663
	A = 3.4 (10)	Own-cat (297)	$\chi_{\left(4\right)}^{2}$ = 4.38	*p* = 0.357
	SA = 2.0 (6)	Aware of Strays (293)	$\chi_{\left(4\right)}^{2}$ = 6.39	*p* = 0.172
Seeing a healthy stray cat would make me feel good (300)	SD = 14.7 (44)	Gender (295)	$\chi_{\left(4\right)}^{2}$ = 3.99	*p* = 0.408
	D = 18.0 (54)	Age (295)	$\chi_{\left(4\right)}^{2}$ = 8.05	*p* = 0.090
	N = 38.3 (115)	Own-pet (297)	$\chi_{\left(4\right)}^{2}$ = 0.80	*p* = 0.938
	A = 17.7 (53)	**Own-cat (300)**	$\chi_{\left(4\right)}^{2}$ **= 20.52**	***p*** < 0.001^***^
	SA = 11.3 (34)	**Aware of Strays (296)**	$\chi_{\left(4\right)}^{2}=$ **14.73**	***p*** = 0.005^**^
Feeding a stray cat would make me feel good (299)	SD = 19.4 (58)	**Gender (291)**	$\chi_{\left(4\right)}^{2}=$ **14.76**	***p*** = 0.005^***^
	D = 14.4 (43)	**Age (294)**	$\chi_{\left(4\right)}^{2}=$ **10.59**	***p*** = 0.032^*^
	N = 34.1 (102)	Own-pet (296)	$\chi_{\left(4\right)}^{2}$ = 0.76	*p* = 0.944
	A = 21.1 (63)	**Own-cat (299)**	$\chi_{\left(4\right)}^{2}$ **= 18.16**	***p*** = 0.001^***^
	SA = 11.0 (33)	Aware of Strays (295)	$\chi_{\left(4\right)}^{2}$ **= 10.52**	***p*** = 0.033^*^
Urban stray cats should be managed differently from feral cats in the bush (302)	SD = 11.3 (34)	Gender (294)	$\chi_{\left(4\right)}^{2}$ = 8.48	*p* = 0.075
	D = 11.3 (34)	Age (297)	$\chi_{\left(4\right)}^{2}$ = 9.17	*p* = 0.057
	N = 28.1 (85)	Own-pet (298)	$\chi_{\left(4\right)}^{2}$ = 5.75	*p* = 0.219
	A = 31.1 (94)	**Own-cat (302)**	$\chi_{\left(4\right)}^{2}$ **= 12.74**	***p*** = 0.013^*^
	SA = 18.2 (55)	**Aware of Strays (298)**	$\chi_{\left(4\right)}^{2}$ **= 18.47**	***p*** = 0.001^***^

* Significant at the < 0.05 level;

** Significant at the ≤ 0.01 level;

*** *Significant at the ≤ 0.001 level. Response distributions associated with significant chi-square results are displayed in plots under the explanation of findings for the given items. Bold indicates variables with significantly different response distributions at P < 0.05*.

**Figure 5:**
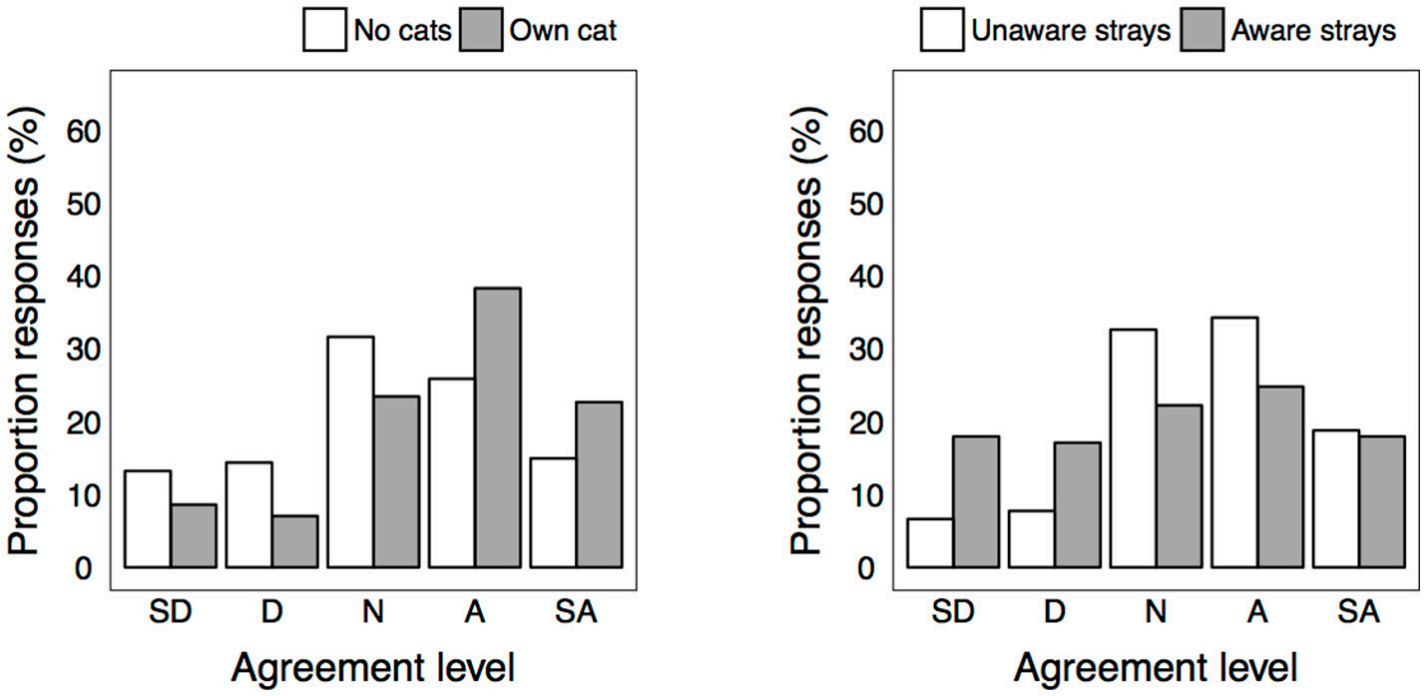
Level of agreement for statement “urban stray cats should be managed differently” for each significantly different group.

More respondents agreed than disagreed that urban stray cats should be managed differently from feral cats in the bush (i.e., forest or wildness areas; 49.3 vs. 22.6%). Cat-owners expressed more agreement than non-cat owners, and those aware of strays expressed more disagreement than those un-aware (Table 4 and Figure 5).

In addition to the stray-welfare items, respondents were asked to decide whether it would be more humane to: (a) euthanize, or (b) leave a stray cat in its environment, if they came across a healthy stray cat in Brisbane. The majority of participants believed it was more humane to leave the cat (71.5%), while others selected euthanasia (27.9%). Respondents were then asked to choose the more humane option if it were the case that they knew the stray cat would die in 2-years-time because it would be hit by a car. After this information, the proportion of respondents who thought it was more humane to leave the cat decreased to 61.0%, while those that believed it was more humane to have the cat euthanized increased to 37.4%.

For the first scenario, chi-square tests revealed that significantly more males, older adults, non-cat owners, and respondents aware of strays selected the euthanasia option than females: $\chi_{\left(2\right)}^{2}$ = 22.93, *p* < 0.001, younger adults: $\chi_{\left(2\right)}^{2}$ = 13.15, *p* = 0.001, cat-owners: $\chi_{\left(2\right)}^{2}$ = 8.41, *p* = 0.016, and those unaware of strays: $\chi_{\left(2\right)}^{2}$ = 24.98, *p* < 0.001. After being told the cat would die, response proportions significantly differed as a function of the same demographic variables described for the first scenario, but the differences were less significant in some cases; gender: $\chi_{\left(2\right)}^{2}$ = 18.54, *p* < 0.001, age: $\chi_{\left(2\right)}^{2}$ = 7.92, *p* = 0.019, cat-ownership: $\chi_{\left(2\right)}^{2}$ = 8.75, *p* = 0.008, awareness of strays: $\chi_{\left(2\right)}^{2}$ = 29.84, *p* < 0.001.

### Managing Urban Stray Cats

Respondents were asked to choose between three alternative options for managing stray urban cats. The first option was: “urban stray cats should be caught, sterilized, microchipped, and vaccinated. Healthy, friendly cats should be adopted to new homes where possible. Those that cannot be found new homes, but are healthy, should be returned to where they were found. Cats that are too sick to be treated should be euthanized (put to sleep).” The second option was to: “continue the current practice of the Brisbane City Council which is to catch ~1,000 stray cats annually in suburban areas (not forests) and to euthanize (put to sleep or kill) most of them.” The third option was to: “leave urban stray cats where they were.” Most respondents (68%) expressed a preference for TNR, while only 28% preferred the current method of managing urban stray cats with culling, and 4% said they should be left alone.

Participants were then provided with information about the efficacy of TNR programs from recent overseas research. Specifically, respondents were informed that: (a) the number of urban stray cats can be reduced by killing them *or* by sterilizing them so that they are unable to have more kittens; (b) to effectively decrease stray cat numbers by killing means that 40% of the population must be killed every 6 months for at least 10 years; (c) in North America and Europe, sterilizing, adopting friendly cats to new homes, and returning the others to where they were found reduces euthanasia of cats and kittens in shelters and pounds, reduces cat-related complaints, and over time, it reduces the number of stray cats in cities at a similar rate as killing cats; (d) that sterilizing and adopting or returning stray cats is often funded by community and welfare agencies, reducing costs to the government compared to killing cats; and lastly, (e) that most urban stray cats are as healthy as owned domestic cats, and less than one in a hundred stray cats (1%) are too unhealthy to be returned to where they were found. After reading this information, respondents were asked again what their preference would be to manage stray cat populations; a greater proportion selected TNR (78%), only 18% selected the current culling method, and 3% elected to leave them alone. The results of a McNemar–Bowker test indicated that the change responses significantly differed to the proportion of responses observed when the same question was answered prior to reading the information supplied, $\chi_{\left(2\right)}^{2}$ = 24.533, *p* < 0.001.

A logistic regression was performed to determine which demographic variables (education level, gender, cat-ownership, age, and SEIFA score) were predictive of respondents' choices for lethal (culling) as opposed to non-lethal (TNR or leaving alone) management strategies for stray cat populations. Option 1 and 3 were collapsed together to create the “non-lethal” option to allow for a binomial logistic regression analysis. The logistic regression was based on management choices in the first question (i.e., prior to receiving information) to gain insight into barriers to TNR support before receiving any persuasive arguments. The model was statistically significant, $\chi_{\left(5\right)}^{2}$ = 33.22, *p* < 0.001 (*n* = 219). It explained 19.6% of the variance in respondents' preferences (Nagelkerke R square), and correctly classified 72.6% of cases (i.e., respondents' preferences). Cat owners were more than three times as likely to select non-lethal management methods than non-cat owners, *p* = 0.001; females were three times more likely to opt for non-lethal methods than males, *p* = 0.001; and an increase in age was associated with an increased likelihood to select lethal, as opposed to the non-lethal management strategies (1.02 times more likely for each increase in age bracket, *p* = 0.019). Education level and SEIFA score were not significant predictors of management preference (*p*s = 0.872 and 0.619, respectively).

A second logistic regression was performed to determine whether respondents' level of agreement to items regarding stray cats' ecological impact (decrease native birds and small animals), risk of disease transmission (spread diseases to humans and pets), and choice of whether leaving or euthanizing a stray cat would be more humane were predictive of stray cat management preferences (lethal vs. non-lethal methods). The model was statistically significant, $\chi_{\left(5\right)}^{2}$ = 118.86, *p* < 0.001 (*n* = 290). It accounted for 48.9% of the variance in management preferences (Nagelkerke R square) and correctly classified 83.8% of cases (i.e., people's preferences). Results indicated that the belief that stray cats spread diseases to humans significantly increased the likelihood of selecting lethal management of stray cat populations. Those that agreed with the statement were significantly more likely to select the culling management option than were those that disagreed (1.60 times more likely for each increase in agreement level, *p* = 0.023). In addition, those that believed it would be more humane to euthanize a stray cat than to leave it in their environment were 14 times more likely to prefer lethal as opposed to non-lethal management than those who thought it would be more humane to leave the cat alone, *p* < 0.001. Opinions as to whether stray cats transmitted diseases to pets, decreased native birds, or decreased native animals did not predict preferences for managing urban stray cat populations (*p*s = 0.587, 0.616, and 0.693, respectively).

When asked whether they would support a trial of TNR in their suburb, in which healthy cats were subsequently adopted or returned to their original location, 71.4% of respondents were in agreement with the suggestion, while 15.6% would not support a trial, and 13.0% were uncertain.

### Knowledge and Opinions About QLD Cat Legislation

Respondents were largely unaware that under Queensland Government law and BCC by-laws there are only two classifications relating to ownership of cats, these being domestic cats (owned by a person) or non-domestic cats (unowned and feral cats). Non-domestic cats are considered “restricted matter” and must not be moved, fed, given away, or sold. Therefore, to feed or adopt urban stray cats or kittens without a permit is not allowed under the Queensland Biosecurity Act 2014^4^ and Land Protection Act 2002 and could result in a fine. Only 11.1% of respondents were aware of these laws.

The majority of respondents (54.8%) disagreed that urban stray cats should be classed as “non-domestic” (feral), while only 28.1% agreed that they should. The remainder (17.1%) did not agree or disagree. Likewise, when asked whether they agreed that urban stray cats must not be moved for adoption, or given away for adoption without a permit, 58.5% disagreed with this, and only 30.5% agreed with the current law. The remainder (11.0%) did not agree or disagree. Finally, 61.4% of respondents disagreed that urban stray cats and kittens should not be fed without a permit, and only 25.9% agreed with the current law. A minority of responders (12.6%) did not agree or disagree.

## Discussion

This study was one of the first to investigate opinions of residents of an Australian city about the problem of urban stray cats. The aim of this study was to explore respondents' experiences and beliefs about urban stray cats and factors associated with negative views toward them. Additionally, this study aimed to investigate preferences for the management of urban stray cats, and factors associated with preferences for lethal as opposed to non-lethal management methods. In doing so, we aimed to identify barriers that need to be addressed to achieve public support for a TNR program to control urban stray cats.

### Sightings, Locations, and Feeding Behaviors of Urban Stray Cats

In the current study, 43% of respondents reported being aware of urban stray cats in their area. There is little information about public awareness of strays in Australian cities, as surveys have tended to focus on relationships between residents and stray cats, and thus specifically aim to sample residents who are aware of strays (6, 10, 30). In an Australian survey of respondents engaged in TNR activities, locations of stray cats most commonly reported were private residences, industrial areas or factory complexes, and streets and alleyways (10). This was similar to the pattern of stray cat sightings in our study, although there was a greater representation of locations such as schools, suburban parks, and commercial businesses. Differences in respondent characteristics and reasons for participating, however, make it difficult to compare between frequencies in these studies.

The proportion of respondents who fed urban strays in the current study (15%) was within the range reported in previous literature. In Australia, 9% of respondents from an internet survey (30), and 22% of Victorian residents in a phone-based survey (59) reported feeding a cat they did not own. In US-based studies, feeding rates of 9% (60), 12% (61), and 26% (26) have been reported. Only 3% of respondents in our study daily fed an unowned cat compared to 9% from an Australian internet survey (30). Findings from published studies suggest that feeders are typically middle-aged and female (26, 30). In the current study, however, similar proportions of males and females fed urban stray cats. More females tend to participate in surveys based on animal welfare than males, which may have resulted in an over-representation of female feeding behaviors in previous studies (30, 62). The current study was distributed to attract an equal proportion of males and females, and although only partially successful, the higher proportion of males than in some studies may account for the differences in feeding demographics than previously observed.

The majority of respondents neither agreed nor disagreed that seeing or feeding a healthy stray cat would make them feel good. Cat-owners however, expressed greater agreement with the statements than non-cat owners, and those aware of strays expressed greater disagreement with the statements than those unaware of strays. For the item, “feeding a stray cat would make me feel good,” it was also found that males and older respondents expressed more disagreement than females and younger respondents. In an Australian study of cat semi-owners (i.e., people who fed cats and provided other care but did not perceive themselves as owners), 87% said feeding a stray cat made them feel good, 76% said “people who are important to me would approve of me feeding a stray cat,” and 58% said “feeding a stray cat is the right thing to do” (6), suggesting that semi-owners derive more satisfaction from caring for a stray cat than is typical for the average population, but similar to cat owners.

### Attitudes and Beliefs Toward Urban Stray Cats

#### Beliefs About Nuisance Behaviors

Respondents' views varied substantially across nuisance behavior items. Interestingly, a large proportion of respondents expressed no opinion toward the items at all. Previous studies have found such behaviors to be a large contributor to the public dislike of strays. In a postal survey study based in Japan, more than a third of respondents reported feces and urine from stray cat colonies being a major nuisance in their community (2). In the United States, loud noises made by cats fighting and the deposition of excrement in communities are common complaints made about urban stray cats (63).

Cat-owners expressed less agreement with the nuisance statements. This is not surprising, as individuals who have a cat or pet are more likely to be understanding and accepting of such behaviors. A California-based study that investigated attitudes toward the fecal deposition of stray cats found that individuals who owned cats themselves were less likely to make complaints about unowned cats, or express concern about health risks related to fecal matter (63). Alternatively, it is also plausible that urban stray cats are less likely to be present around properties of those who own cats, if the domestic cats spend time outdoors around the property and defend their territory. In Australia in 2016, 62% of households owned a pet, and 29% owned a cat (64). The comparatively larger proportion of pet (76%) and cat owners (56%) in our study may have contributed to the lower level of negativity toward urban stray cats for these behaviors than reported in prior literature.

Older respondents and those that were aware of strays expressed more negative views toward urban stray cat nuisance behaviors than younger respondents and those unaware of strays. In gaining public support for a community-based TNR program, arguments that are likely to be persuasive to these individuals should emphasize the efficacy and viability of TNR for reducing stray cat populations, which in turn would result in a reduction in the prevalence of such nuisance behaviors.

#### Beliefs About Disease Spread

Only 18% of respondents agreed that urban stray cats spread disease to humans, while 39% disagreed. This relatively low level of concern might reflect the large proportion of cat-owners in our study. Cat-owners were less concerned about the risk of disease transmission than non-cat owners in our study, which aligns with previous findings (63). Respondents who were aware of strays perceived a higher risk of disease transmission than those unaware, however, these respondents had a more negative impression of stray cats in general, with consistently more negative views about stray cats across every survey item tested.

A review of feral cat management strategies has listed the risk of zoonotic diseases as a major cause of public concern regarding stray cats in the United States (1). Articles about TNR programs commonly cite public concern about disease spread as a significant contributor to the opposition of TNR programs (36, 51). Several diseases are of concern, including toxoplasmosis, ringworm, bartonella, and rabies (65). Most are spread by direct contact or fleas, except toxoplasmosis, and rabies does not occur in Australia. Contrary to concern expressed by respondents in our and other studies, there is a low risk of disease transmission from cats to humans (66), and for most diseases, the risk of transmission is even lower from stray cats due to the lack of direct contact. Diseases transmitted from cats are much more likely to come from pet cats who are more frequently in contact with the general public.

Concerns are often raised about toxoplasmosis, which for most healthy humans results in no clinical signs. However, in humans with weakened immune systems or pregnant women, toxoplasmosis can cause serious disease (65). Although infection can occur from accidentally ingesting cat feces with oocysts (eggs) from contaminated hands, especially in children, most infections are caused by the handling or ingestion of poorly cooked/uncooked meat; toxoplasmosis can infect sheep, cattle, pigs and wildlife (65). There is no association between cat ownership and the presence of toxoplasmosis antibodies indicating human exposure (67, 68). Furthermore, environmental contamination with toxoplasmosis oocysts is likely reduced in TNR programs compared to trap and kill programs. This is because the average age of cats in TNR programs is higher than in trap and kill programs; older cats are more likely to be immunized from previous exposure and usually they do not become infected or shed oocysts in feces after the initial infection (46, 48). In contrast, in trap and kill programs, young immunologically naïve kittens are continuously being born, get infected, and shed oocysts in feces. Immunologically naïve cats older than 1 year, if infected, shed fewer oocysts than cats younger than 1 year (47). Educating the public about the actual level of disease transmission risk, and that it is further reduced with TNR, may help to improve impressions of urban stray cats in communities, and lead to more public support of a community TNR program.

More respondents agreed that stray cats spread diseases to pet cats (48%) than to humans (18%), and indeed cellulitis and abscessation resulting from cat scratches or fights is a common occurrence in pet cats with outdoor access (69). However, for potentially fatal infectious diseases, stray cats have similar or lower prevalence rates of infections than those published for pet cats in the United States (36, 70) and the prevalence of feline leukemia virus (FeLV) and feline immunodeficiency virus (FIV) were lower in shelter cats than owned cats with outdoor access in Australia (71). Disease transmission is reduced once cats are sterilized for diseases such as FIV which are spread by fighting.

#### Beliefs About Ecological Impact

Respondents' views concerning the impact of stray cats on local wildlife widely varied. A higher proportion of respondents agreed (32%) that stray cats negatively affected wildlife than those that disagreed (18%). Concern over wildlife predation and the impact of cats on sensitive ecosystems has traditionally been one of the major problems leading to negative perceptions of cats in Australia (8, 28, 51). In a recent study that investigated attitudes toward wildlife predation by pet cats across different countries, Australians expressed the most extreme attitudes toward pet cats' impact on native wildlife in comparison to other countries. Surveys were distributed to two cities in each of the 6 countries included. Results demonstrated that 95% of Australian non-cat owners and 65% of cat owners agreed that pet cats posed a serious threat to animals and the environment (8).

The same recurring trend in responses emerged for this item whereby cat-owners expressed less negative views about the ecological impact of stray cats than non-cat owners, and those aware of strays in their area expressed more negative views than those unaware of strays. Interestingly however, females were seen to express more disagreement with the ecological impact items than males. It is unclear why differences in ecological impact beliefs may arise as a function of gender. Previous literature indicates that females are more compassionate than males toward animals (72). Perhaps as a result of this they are less forthcoming in placing blame on urban stray cats.

Previous literature has shown that concern about the ecological impact of stray cats differs depending on the location of respondents (urban vs. rural). A study conducted in Japan found that stray cats were perceived more positively in urban areas compared to stray cats that inhabited forests or wilderness areas home to endangered species (29). Additionally, a trend in the international ecological impact survey was evident whereby the strongest attitudes were observed in countries with the greatest endemic biodiversity (8). This aligns with findings from a study conducted in the United States, that found that the popularity of lethal stray cat population management increased as town/city size decreased (28). It is possible that the high proportion of neutral responses to environmental impact questions in the current study could be reflective of respondents coming from relatively low biodiversity suburban areas.

#### Beliefs About Welfare

Overall, findings demonstrated that very few Brisbane City residents (5.4%) thought stray cats lead a good life, and a substantial proportion (27.4–37.0%) believed that euthanizing an urban stray cat would be more humane than leaving it in its environment. This is higher than a previous study in the United States where 14 and 21% of respondents elected euthanasia in response to the same question (3). Results suggested that females and younger respondents may place more value on the lives of urban stray cats than males and older respondents, in that they less frequently selected the euthanasia option.

Contrary to respondent's views, urban stray cats are documented to have health and welfare scores comparable to that of owned pet cats (36, 70). Several studies have found that < 1% of cats coming into TNR programs had health problems significant enough to warrant euthanasia (37, 73–75). In addition, the welfare of urban stray cats in colonies managed by TNR was not different from pet cats (75). Misconceptions of stray cat welfare have been proposed to contribute to less favorable opinions of TNR programs (36). As found in our study, preferences amongst US respondents for lethal population control were strongly associated with the perception that euthanasia would be more humane (51). However, it is possible that some respondents' choices were motivated by a preference for culling rather than perceptions of comparative humaneness, as it is likely that some respondents did not have any regard for stray cat welfare. Public education programs intended to foster community support for TNR should focus on dispelling negative beliefs about stray cats' welfare that are not backed up by evidence, and emphasize the efficacy of TNR to reduce issues linked to cat-related complaints.

### Preferences for Managing Urban Stray Cats

Most respondents recognized that urban stray cats are not the same as feral cats, and accordingly they should be managed differently to feral cats in the bush (only 22.6% disagreed with the statement). Those that were aware of strays in their area, however, showed greater disagreement with this statement, which may indicate that they are more likely to equate urban stray cats to feral cats in the bush. Few respondents (11%) were aware that Queensland legislation classified urban stray cats as “restricted matter” which must not be moved, fed, given away, or sold. However, it should be noted that items pertaining to knowledge of Queensland legislation, and whether respondents agreed with this legislation, were responded to in a yes/no format. This limits the interpretation, because it is unknown whether respondents may have known some aspects of the law. Therefore, it is difficult to make strong inferences about these results. That said, survey respondents did have the opportunity to share any additional information or views in a written format at the end of the survey.

The majority of respondents supported a TNR community program as their preferred method for managing urban stray cats (78%). A smaller but substantial portion selected culling (18.1%), and a very small portion chose to leave the cats alone (3.4%). Information about the effectiveness and welfare of cats in TNR community programs lead to a modest but significant increase in support for TNR (from 68 to 78%). Although it was evident that the majority of respondents were in favor of TNR as an effective means of stray cat population management, it is important to explore reasons why other respondents did not support a TNR community program.

#### Predictive Demographic Variables

Respondents were more likely to select lethal means of stray cat management if they were male, of an older age, and if they were non-cat owners. The association between gender and management preference aligns with findings from an Ohio-based study that also reported male gender being associated with a greater preference for culling rather than a TNR program (26). Prior literature demonstrates that women show greater concern and compassion toward the welfare of animals than men, and are more emotionally disturbed by mistreatment such as unnecessary killing (28, 76). Generational differences may underpin the association between older adults and lethal management preferences; one study has argued that younger individuals are more likely to show pro-animal welfare attitudes (76). In a more recent study, however, little association between age and attitudes toward animals was found (77). Furthermore, previous literature on TNR attitudes has demonstrated that non-cat owners are not as supportive about TNR programs as cat owners (2, 51).

Overall, what has been observed in survey responses suggests that older adults, males, and non-cat owners have less concern about the welfare of stray cats in general, and as a result, it is not likely that these groups would be persuaded by arguments that highlight the humanitarian merit of TNR as an alternative to culling. Instead, appealing to the practicality of TNR over culling is likely to be more persuasive for these groups. Hence, information should more heavily focus on the comparison of implementation costs and viability between a TNR program and a large-scale culling program, the decrease in stray cat populations and stray cat-related complaints, as well as, the reduced risk of disease transmission from stray cats to humans, wildlife or pets after implementing a TNR program. Information should also generate awareness of the mental health damage to shelter workers euthanizing kittens and cats, and that fewer numbers are required to be euthanized in TNR programs.

The information provided to participants in our survey did not explicitly compare the efficacy of culling compared to a TNR program, but reported that culling *or* TNR can be effective at reducing stray cat populations. It was also stated that the TNR programs trialed have been able to reduce stray cat populations as effectively as culling. It was not made clear, however, that the TNR efficacy was being compared to a calculated, large-scale culling practice instead of the current Brisbane City Council culling practices that are not effective in decreasing overall cat numbers in the medium to long-term, or evidence-based. Hence, the persuasive information could have been presented more clearly to outline the practical benefits of a TNR program. Other benefits that were clearly stated included that friendly stray cats and kittens would be able to be adopted and re-homed, and euthanasia in shelters and pounds would decrease as a result of TNR. While it is an extremely important and positive consequence of TNR, the argument is not likely to have been effective for these groups given their views on urban stray cats. Based on observed attitudes and the content of the persuasive information provide to participants, it is unsurprising that a more substantial increase was not achieved in the proportion of respondents that selected TNR.

#### Predictive Beliefs

Respondents were more likely to select lethal means of stray cat management if they believed that stray cats spread diseases to humans. Although there were only a small proportion of respondents that expressed this belief, it is evident that it had a strong impact on the selection of preferred stray cat management strategies. Furthermore, lethal management methods were significantly more likely to be preferred by respondents who believed euthanizing a stray cat would be more humane than leaving it in their environment.

Residents that believe stray cats pose a serious health risk to humans are unlikely to support a program that releases stray cats back into the environment, or stipulates that sociable cats be adopted. In the passage of information provided to respondents outlining the merits of TNR, there was no mention of disease transmission risk, or the welfare of urban stray cats. It was stated that urban stray cats have health comparable to that of owned-pets, and that they are rarely too unhealthy to be returned to where they were found. If the information had included a section that addressed concerns relating directly to disease transmission risks and cat welfare, a more substantial increase in the proportion of respondents selecting TNR as the preferred cat-management strategy may have been observed. It is important that information outlining the benefits of TNR over lethal population management strategies firmly and directly addresses risk of disease transmission, and highlights the good welfare of most urban stray cats to dispel the notion that euthanasia would be humane.

### Limitations

Some limitations should be acknowledged when interpreting findings from this study. Firstly, although the sampling method was specifically designed to target a representative sample of respondents, the sample was predominantly female (70%), and therefore the data is more reflective of a female perspective. Education level and cat-ownership status also deviated from that of the general population, with our sample being comparatively more educated [48% had a bachelor degree vs. 31% in Australian population; (78)], and consisting of more cat-owners [49 vs. 29%; (64)]. Cat-owners have been observed to hold more positive views toward stray cats, though prior studies suggest that TNR preferences are not influenced by education level (28). The higher proportion of females and cat-owners should be considered when generalizing these findings to the wider Australian population. As noted, some survey questions did not allow for a detailed response (i.e., invoked yes/no answers), and therefore inferences that can be made are limited. Lastly, the information covering stray cat management strategies could have been presented more clearly, which may have led to a more compelling response.

### Implications for Policy and Further Research

Results from this study demonstrated that current Queensland legislation does not align with the beliefs or preferences of Brisbane City residents. Only a small minority of respondents agreed that urban stray cats should be classed as “feral” and must not be adopted or fed. Most Brisbane City residents indicated that TNR was their preferred method for managing urban stray cats rather than the current Brisbane City Council method of culling, and an overwhelming proportion supported a trial of TNR for urban stray cats in their suburb. Conducting trials of TNR in urban areas of Australia where stray cats are a source of complaints, or overrepresented in shelter intake, are needed to provide evidence for the efficacy and viability of TNR over current practices.

## Conclusion

Results of this study have shown that for most Brisbane City residents, when awareness is raised about the problem of urban stray cats and management strategies, the majority are supportive of a TNR community program with little or no persuasion required. Results have illuminated that certain groups—males, older adults, non-cat owners, and those aware of strays—are less easily persuaded about the merits of TNR. Findings from this study indicate that appealing to the practicality of TNR is likely the optimal strategy in disseminating information that will appeal to all demographic groups. Specific concerns or negative beliefs about stray cats can be targeted by emphasizing the efficacy in steadily reducing populations of urban stray cats, and in turn, the nuisances associated with them. In addition, this study brought to light harmful and erroneous beliefs that information promoting TNR should dispel in order to achieve public support. Beliefs about disease transmission and the humanness of euthanasia were significant predictors of lethal management preferences, and negative beliefs about urban stray cats' welfare were widespread. Information disseminated about TNR needs to address the health and wellbeing of urban stray cats, and the low risk of disease transmission. In conclusion, this study pinpointed the beliefs and demographic variables associated with negative views about stray cats and TNR, and has provided clear recommendations for the type of information to be disseminated to combat such barriers.

## Ethics Statement

This study adheres to the Guidelines of the ethical review process of The University of Queensland and the National Statement on Ethical Conduct in Human Research. All subjects were provided with an information sheet about the survey prior to participation in the study. Participants indicated their consent by checking a box that read I understand that this survey is about managing stray cats and I agree to participate (please tick).

## Author Contributions

JR conceived the experiment. JR, KL, and AH designed the experiments. GF analyzed the data. KL wrote a draft of the pilot data which was extensively reworked by GF and JR with input from AH.

### Conflict of Interest Statement

The authors declare that the research was conducted in the absence of any commercial or financial relationships that could be construed as a potential conflict of interest.

## Footnotes

^1^Brisbane City Council (BCC). Biodiversity in Brisbane. Available online at: https://www.brisbane.qld.gov.au/environment-waste/natural-environment/biodiversity-brisbane

^2^Queensland Government 2014. Queensland Biosecurity Act 2014. *In*: Government, Q. (ed.). Brisbane.

^3^Queensland Government 2002. Land Protection (Pest and Stock Route Management) Act 2002. *In*: Government, Q. (ed.). Brisbane.

^4^Queensland Biosecurity Act 2014. (2014).
