# Supplementary material for: Public Opinions on Strategies for Managing Stray Cats and Predictors of Opposition to Trap-Neuter and Return in Brisbane, Australia
Source: Front Vet Sci. 2019 Feb 18;5:290. doi: 10.3389/fvets.2018.00290 (PMC6387915; doi:10.3389/fvets.2018.00290)
Supplement: Supplementary file 1 [file Data_Sheet_1.pdf]

Appendix

Table A1: Managing Stray Cats in Brisbane Survey

Preamble:

The purpose of this survey is to gauge community attitudes towards various methods of managing stray cats (cats and kittens with no known owner and living within suburbs). Your participation is vital to the success of this project. The results of the study will be published in an international journal. Participation in this research is voluntary. Your confidentiality is assured and no responses will be linked to you individually. The survey should take approximately 10 minutes to complete. You must be at least 18 years of age to participate.

Survey:

1. Are you aware of urban stray cats with no known owner and living within suburbs, at any of the following areas? Please select all that apply. If not aware of any stray cats, please choose the last option and proceed to **Question 3.**

I am aware of stray cats:

- Near where I live
- Near where I work, study, or where I spend most of my time when away from home
- I am not aware of any stray cats near where I live, work, study, or spend most of my time when away from home.

2. If you are aware of urban stray cats (cats with no known owner) in any of these areas near where you live or spend most of your time when away from home, please select all locations that apply. If known, add suburb or postcode for area by writing it in next to the location you've selected.: Industrial areas

- School or university	-Train or bus station
- Shopping center	-Food shops or restaurants
- Hospital	-Private residence
- Suburban Park	-Alley ways or street
- Government housing	-Vacant blocks or vacant building
- Other (please specify)	

3. Please indicate whether you agree or disagree (Strongly disagree; disagree; neither disagree or agree; agree; strongly agree; don't know) with the following statements:

Urban stray cats cause a nuisance by urinating (peeing) and defecating (pooing) in people's gardens

- Urban stray cats are annoying because they fight and make loud noises
- Urban stray cats spread diseases to humans
- Urban stray cats spread diseases to owned pets
- Urban stray cats have a good life
- Urban stray cats should be managed differently from feral cats in the bush

4. If you saw a healthy stray cat in Brisbane, and could only choose between two courses of action —leaving the cat where it is outside, or having the cat caught and then euthanased (put down)—which would you consider to be the more humane option for the cat? (select only one answer)

- Leave the cat where it is
- Have the cat euthanased (put to sleep, killed) now

5. If you knew that the healthy stray cat you saw in Brisbane would die in two years because it would be hit by a car, which would you consider the most humane option today? (select only one answer)

- Leave the cat where it is, and let it live two years before dying
- Have the cat euthanased (put to sleep) now

6. In the last 12 months, have you ever fed a cat that does not belong to you, or anyone you know? If **no**, go to **Question 8**.

7. If yes, how often do you feed the cat(s)?
Daily; A few times a week; A few times a month; A few times a year

8. Please indicate whether you agree or disagree (strongly disagree; disagree; neither disagree or agree; agree; strongly agree; don't know) with the following statements:

- Urban stray cats have decreased the number of native birds in my suburb
- Urban stray cats have decreased the number of small native animals in my suburb
- Urban stray cats in my suburb kill mice and rats
- Seeing a healthy stray cat makes me feel good
- Feeding an urban stray cat makes me feel good

9. At this moment, what would be your preference among these three options for managing stray cats in Brisbane suburbs (not in bushland areas)? (select only one answer)

- Option 1: Urban stray cats should be caught, sterilized, microchipped and vaccinated. Healthy, friendly cats should be adopted to new homes where possible. Those that cannot be found new homes, but are healthy, should be returned to where they were found. Cats that are too sick to be treated should be euthanased (put to sleep)
- Option 2: Continue the current practice of the Brisbane City Council which is to catch approximately 1000 stray cats annually in suburban areas (not forests) and to euthanase (put to sleep or kill) most of them
- Option 3: Urban stray cats should be left alone where they are

Please add any additional comments:

10. Please consider the following findings from recent research on urban stray cats:

- The number of urban stray cats can be reduced by killing them, or by sterilizing them so that they are unable to have more kittens.
- To effectively decrease stray cat numbers by killing means that 40% of the population must be killed every 6 months for at least 10 years.

- In North American and Europe, sterilizing, adopting friendly cats to new homes, and returning the others to where they were found, reduces euthanasia of cats and kittens in shelters and pounds, and reduces cat-related complaints. And over time, it reduces the number of stray cats in cities at a similar rate as killing cats.
- Sterilizing and adopting or returning stray cats is often funded by community and welfare agencies, reducing costs to government compared to killing cats.
- Most urban stray cats are as healthy as owned domestic cats, and less than one in a hundred stray cats (1%) are too unhealthy to be returned to where found.

Knowing these research findings, what would be your preference NOW for managing urban stray cats in Brisbane? (select only one answer)

- Option 1: Urban stray cats should be caught, sterilized, microchipped and vaccinated. Healthy, friendly cats should be adopted to new homes where possible. Those that cannot be found new homes, but are healthy, should be returned to where they were found. Cats that are too sick to be treated should be euthanased (put to sleep)
- Option 2: Continue the current practice of the Brisbane City Council which is to catch approximately 1000 stray cats annually in suburban areas (not forests) and to euthanase (put to sleep or kill) most of them
- Option 3: Urban stray cats should be left alone where they are

11. Please indicate if you would support a trial or pilot project in your suburb where urban stray cats are caught, sterilized (sterilised and unable to reproduce), vaccinated and microchipped. Healthy, friendly cats are adopted to new homes where possible. Other healthy cats would be returned close to they were found. Cats that are too sick to be treated are euthanased (put to sleep). Yes / No / Unsure

12. Under Queensland Government law and Brisbane City Council by-laws there are only two classifications relating to ownership of cats, these being domestic cats (owned by a person) or feral cats (without an owner). Feral cats must not be moved, fed, given away, or sold. Therefore, to feed or adopt urban stray cats or kittens without a permit is not allowed under the Qld Biosecurity Act 2014, and could result in a fine. Did you know this? Yes / No

13. Do you agree or disagree (Strongly disagree; disagree; neither disagree or agree; agree; strongly agree; don't know) that unowned "stray" cats in urban areas should be classed as "feral"? This means that under Queensland Government law and Brisbane City Council bylaws stray cats must not be moved for adoption, fed, given away to someone for adoption or sold, without a permit.

- Urban stray cats and kittens should be classed as feral
- Urban stray cats and kittens must not be moved for adoption or given away for adoption without a permit
- Urban stray cats and kittens must not be fed without a permit

14. What is your age

-18-24 -25-29 -30-34 -35-39 -40-44 -45-49 -50-54
-55-59 -60-64 -65-69 -70-74 -75-79 -80-84

15. What is the postcode where you live?

16. What is your gender? Male / Female / Other

17. What country were you born in?

18. What language is mostly spoken in your home?
English / Other

19. How would you describe where you live?
-I pay rent -I live in a shared house
-I own my house -I live with a family member
-I am a visitor where I am staying

20. What is your highest level of education?
-Secondary School -Certificate or Diploma
-Bachelor degree -Graduate Diploma
-Postgraduate degree

21. Do you own a pet?
Yes / No If no, please go to **Question 26**

22. If yes, what pet/pets do you own? Tick all that apply.
-Cat/s -Dog/s -Bird/s -Fish -Reptile/s -Other (please specify)

23. If you own any cats, how many do you own?
If you do not own a cat, please proceed to **Question 26**

24. Are your cats microchipped?
-Yes, all microchipped -None are microchipped
-Some yes, some no -Don't know

25. Are your cats sterilized (i.e. spayed, neutered, sterilized and unable to reproduce)?
Yes / No / Some yes, some no / Don’t know

Additional comments regarding stray cats (if needed, extra space for comments over page):

Thank you for your time.
A summary of the results of this survey will be published in an international scientific journal, and provided to the Queensland Government and Brisbane City Council.

Table A2. Association between beliefs about nuisance behaviors and demographic factors

Demographic attribute and attitudes to cats		Gender (n=294)		Age (yr.)		Pet Owner		Own cat		See stray cats	
		F	M	<40	≥40	Y	N	Y	N	Y	N
Find cats defecating annoying	Agree	44% (93)	52% (43)	**37% (63)**	**57% (72)**	**43% (97)**	**54% (38)**	**30% (38)**	**57% (99)**	**59% (69)**	**36% (66)**
	Disagree	30% (64)	20% (16)	**33% (56)**	**21% (27)**	**32% (73)**	**16% (11)**	**46% (58)**	**15% (27)**	**23% (27)**	**31% (57)**
	Uncertain	26% (55)	28% (23)	**30% (51)**	**22% (28)**	**25% (58)**	**30% (21)**	**24% (31)**	**28% (49)**	**18% (21)**	**32% (58)**
Find cats fighting annoying	Agree	43% (92)	54% (44)	**35% (60)**	**61% (78)**	45% (102)	50% (35)	**32% (41)**	**57% (99)**	**56% (65)**	**40% (73)**
	Disagree	29% (62)	15% (12)	**31% (52)**	**19% (24)**	28% (64)	17% (12)	**40% (51)**	**15% (26)**	**22% (25)**	**28% (51)**
	Uncertain	27% (58)	32% (26)	**34% (58)**	**20% (25)**	27% (62)	33% (23)	**28% (36)**	**28% (49)**	**22% (26)**	**32% (58)**
Believe cats spread diseases to pets	Agree	47% (99)	52% (42)	47% (81)	49% (60)	49% (111)	44% (30)	**41% (53)**	**53% (91)**	**59% (67)**	**42% (76)**
	Disagree	19% (39)	12% (10)	15% (26)	21% (26)	19% (44)	12% (8)	**27% (35)**	**10% (17)**	**14% (16)**	**19% (35)**
	Uncertain	34% (72)	36% (29)	37% (64)	30% (37)	32% (72)	44% (30)	**31% (40)**	**37% (63)**	**27% (30)**	**39% (71)**
Believe cats spread diseases to humans	Agree	17% (35)	22% (18)	18% (30)	19% (24)	**18% (41)**	**17% (12)**	**7% (9)**	**26% (45)**	**27% (31)**	**12% (22)**
	Disagree	40% (84)	32% (26)	39% (66)	38% (47)	**44% (100)**	**20% (14)**	**61% (77)**	**22% (38)**	**39% (45)**	**38% (69)**
	Uncertain	43% (91)	46% (38)	44% (75)	43% (53)	**38% (86)**	**62% (43)**	**32% (41)**	**52% (90)**	**34% (39)**	**50% (91)**
Believe cats decrease local birds	Agree	**27% (56)**	**48% (39)**	29% (48)	36% (46)	33% (74)	30% (21)	**27% (34)**	**36% (62)**	**51% (58)**	**20% (37)**
	Disagree	**20% (41)**	**10% (8)**	20% (33)	13% (17)	19% (43)	10% (7)	**26% (33)**	**10% (18)**	**15% (17)**	**18% (33)**
	Uncertain	**54% (112)**	**42% (34)**	51% (85)	50% (64)	48% (108)	60% (42)	**47% (59)**	**53% (92)**	**34% (38)**	**61% (111)**
Believe cats decrease local animals	Agree	**27% (57)**	**51% (41)**	37% (48)	57% (49)	33% (75)	33l% (23)	**26% (33)**	**38% (66)**	**54% (60)**	**21% (38)**
	Disagree	**20% (42)**	**11% (9)**	33% (34)	21% (18)	20% (44)	11% (8)	**27% (34)**	**11% (19)**	**15% (17)**	**19% (35)**
	Uncertain	**52% (109)**	**38% (31)**	30% (83)	22% (60)	47% (105)	56% (39)	**46% (58)**	**51% (87)**	**31% (35)**	**60% (108)**

Items in bold are significantly different at the P<0.05

Table A3. Factors influencing choice of future options for managing stray urban cats (choices are prior to respondents being provided with information on the efficacy and welfare of TNR programs)

Demographic attribute and attitudes to cats		Option 1: Support a TNR program	Option 2: Euthanize urban stray cats	Option 3: Leave cats alone
**Gender**	**Female**	**76% (160)**	**21% (44)**	**3% (7)**
	**Male**	**45% (37)**	**48% (39)**	**7% (6)**
**Age (yr.)**	**<40**	**72% (121)**	**24% (41)**	**4% (7)**
	**≥40**	**63% (80)**	**32% (41)**	**5% (6)**
Own Pets	Yes	70% (159)	25% (58)	5% (11)
	No	62% (43)	35% (24)	3% (2)
**Have pets other than cats**	**Yes**	**56% (55)**	**39% (39)**	**5% (5)**
	**No (Cat Owners)**	**74% (150)**	**22% (44)**	**4% (8)**
Birds and no Cats	Yes	40% (6)	60% (9)	0% (0)
	No	70% (199)	26% (74)	5% (13)
Have seen stray cats	Yes	57% (65)	36% (41)	7% (8)
	No	75% (137)	22% (41)	3% (5)
Feed stray cats	Yes	79% (34)	12% (5)	9% (4)
	No	67% (169)	30% (76)	4% (9)
Believe cats bring nuisance by urinating (n=163)	Agree	51% (68)	46% (62)	3% (4)
	Disagree	87% (74)	9% (8)	4% (3)
	Uncertain	76% (60)	16% (13)	8% (6)
Believe cats bring annoyance by fighting (n=163)	Agree	53% (73)	43% (60)	4% (5)
	Disagree	89% (68)	7% (5)	4% (3)
	Uncertain	74% (62)	20% (17)	6% (5)
**Believe cats spread diseases to humans**	**Agree**	**45% (24)**	**53% (28)**	**2% (1)**
	**Disagree**	**82% (94)**	**12% (14)**	**5% (6)**
	**Uncertain**	**65% (85)**	**30% (39)**	**5% (6)**
Believe cats bring disease to pets (n=162)	Agree	61% (87)	36% (51)	3% (5)
	Disagree	88% (45)	10% (5)	2% (1)
	Uncertain	69% (70)	25% (25)	7% (7)
Believe cats decrease local birds	Agree	52% (49)	48% (46)	0% (0)
	Disagree	82% (41)	8% (4)	10% (5)
	Uncertain	74% (111)	20% (30)	5% (8)
Believe cats decrease local animals	Agree	53% (51)	46% (45)	1% (1)
	Disagree	88% (46)	4% (2)	8% (4)
	Uncertain	72% (104)	22% (32)	6% (8)

Items in bold are significantly different at the P<0.05
